# Antibody-Mediated Extreme Insulin Resistance: The Importance of Pre-Treatment Screening

**DOI:** 10.1155/2019/8562546

**Published:** 2019-11-16

**Authors:** Tina Mosaferi, Sahar Sherf, Laura Y. Sue, Ines Donangelo

**Affiliations:** ^1^Department of Medicine, David Geffen School of Medicine, University of California Los Angeles, Los Angeles, CA, USA; ^2^Division of Endocrinology, David Geffen School of Medicine, University of California Los Angeles, Los Angeles, CA, USA

## Abstract

We report the case of a 56 year-old Hispanic male with a 10-year history of type 2 diabetes who presented with abrupt onset of hyperglycemia resistant to escalating doses of intravenous insulin infusion (>2500 units daily). He was diagnosed with antibody-mediated insulin resistance given the presence of hyperglycemia despite receiving >200 units insulin/day, a lack of identifiable precipitants for diabetic ketoacidosis or hyperosmolar hyperglycemic state, and elevated insulin antibodies. He underwent pre-immunomodulatory therapy screening for infections, rheumatologic disorders, and malignancy, which uncovered a new diagnosis of latent tuberculosis. While concurrently being treated for latent tuberculosis, he successfully responded to immunomodulatory therapy with rituximab, dexamethasone, and cyclophosphamide. Insulin was discontinued completely, and he maintained appropriate glycemic control on oral diabetic agents (metformin and pioglitazone). This case supports the use of immunomodulatory therapy for the treatment of antibody-mediated insulin resistance and highlights the importance of pre-immunomodulatory therapy screening to uncover occult infection or identify underlying neoplastic/rheumatologic disease prior to immunosuppression.

## 1. Introduction

Antibody-mediated extreme insulin resistance is characterized by hyperglycemia despite the use of >200 units of insulin/day and is often divided into two subtypes, insulin receptor antibody-mediated (Type B insulin resistance) and insulin antibody-mediated insulin resistance. Although the exact prevalence remains unknown, concern for high mortality has been reported [1]. The National Institutes of Health (NIH) published an immunomodulatory protocol for the treatment of Type B insulin resistance in 2010 [2], yet little has been documented to guide pretreatment screening. This article aims to present a case of antibody-mediated insulin resistance that highlights the challenge of subtype diagnosis and emphasizes the importance of pretreatment screening.

## 2. Case Report

A 56 year-old Hispanic male with hypertension and a 10-year history of diabetes was hospitalized for abrupt worsening of glycemic control and diabetic ketoacidosis (DKA). His diabetes was initially managed on metformin and sitagliptin, with insulin added six years after diagnosis. His glycemic control had been stable with home self-monitored blood glucoses (SMBG) averaging around 160 mg/dL, however there was an abrupt rise in SMBG to around 400 mg/dL starting six months prior to presentation. After hospitalization at another institution two months prior for DKA, he was discharged on detemir insulin 20 units every morning, detemir insulin 40 units every evening, and aspart insulin 15 units before each meal.

**Figure 1:**
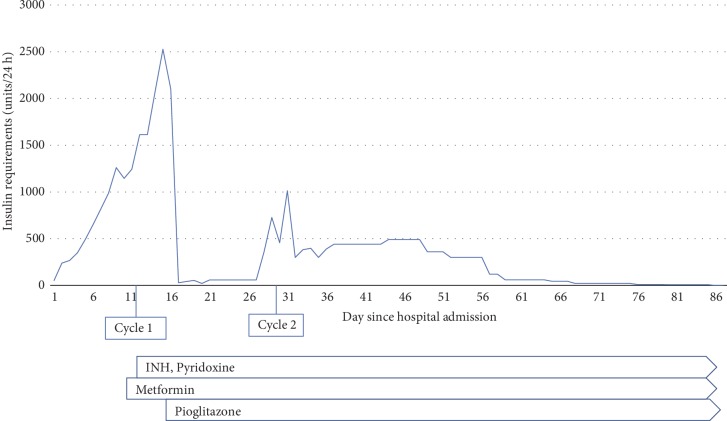
Clinical course defined by daily insulin requirements and initiation of oral antihyperglycemic therapy, diagnosis of latent tuberculosis, and initiation of immunomodulatory therapy. Cycle 1 of immunomodulatory therapy was initiated concomitantly with latent tuberculosis treatment.

He presented to our emergency department with 18-kg weight loss, initial blood glucose 427 mg/dL, and a hemoglobin A1c 12.4% (112 mmol/mol) with bicarbonate 15 mmol/L (20–30 mmol/L), anion gap 18, moderate serum ketones, and 2+ urine ketones. On physical exam he weighed 57.9 kg, with a BMI of 24.93 kg/m^2^. He was normotensive with regular heart rate. No acanthosis nigricans, dorsoclavicular fat pad, abdominal striae, or skin lesions were present. He was started on intravenous regular insulin infusion. Despite prompt normalization of the metabolic acidosis, he continued to have markedly high insulin needs to keep serum glucose levels between 150–250 mg/dL. The peak insulin infusion rate was 2,526 units/24 hours (Figure 1). There was no meaningful reduction in daily insulin requirements that occurred with the addition of metformin 500 mg oral twice daily or concentrated U-500 regular insulin (up to 300 units before each meal). Adiponectin level was normal at 18 mcg/mL (4–20 mcg/mL) with intact C-peptide of 3.0 ng/mL (1.1–4.3 ng/mL) and concomitant serum glucose level of 226 mg/dL. He was diagnosed with antibody-mediated insulin resistance with evidence of elevated insulin antibody levels (12.4 U/mL, normal range 0.0–0.4 U/mL). No commercial assay was available to measure the insulin receptor antibody level. Despite being unable to confirm the presence of insulin receptor antibodies, the decision was made to initiate the NIH treatment protocol for Type B insulin resistance [2, 3], which includes rituximab, dexamethasone, and cyclophosphamide. Evaluation for malignancy, rheumatologic disease, and infection was performed prior to initiating immunosuppression. Computed tomography (CT) of the chest, abdomen, and pelvis was negative for malignancy. Serum and urine protein electrophoresis was negative for monoclonal gammopathy, and flow cytometry was negative for lymphoproliferation. Prostate specific antigen was normal at 0.44 ng/mL (0–3.5 ng/mL). Autoimmune workup revealed mildly elevated antinuclear antibody titer (1 : 80, normal <1 : 40) and double stranded DNA antibody level (220 IU/mL, normal ≤200 IU/mL) but was otherwise unremarkable (negative for glutamic acid decarboxylase-65, thyroid peroxidase, SSA, SSB, smith, smooth muscle, centromere B, cyclic citrulline, RNP, scleroderma, and Jo 1 antibodies). Infectious workup revealed positive *Mycobacterium tuberculosis* Quantiferon-GOLD TB-Nil > 10 IU/mL (positive ≥0.35 IU/mL), but subsequent acid-fast culture and stain and respiratory *Mycobacterium tuberculosis* PCR were negative. CT of the chest did not show evidence of active tuberculosis. Remaining infectious disease studies were negative (Hepatitis B core Ig, Hepatitis B surface antigen, Hepatitis C antibody, Hepatitis A IgM, HIV-1/2 Ag/Ab, strongyloides IgG, coccidioides IgG/IgM, and urine histoplasma antigen). After initiation of treatment for latent tuberculosis with isoniazid and pyridoxine, he underwent cycle 1 of the NIH immunomodulatory protocol with marked reduction in daily insulin requirements (Figure 1). Pioglitazone 45 mg daily was then initiated. One month following two treatment cycles, insulin therapy was discontinued and blood glucose levels were controlled on metformin and pioglitazone alone (Figure 1).

## 3. Discussion

The abrupt, marked insulin resistance in this case drew suspicion for an autoimmune etiology and antibody-mediated insulin resistance was ultimately diagnosed. We were unable to further distinguish between Type B insulin resistance and insulin antibody-mediated insulin resistance. Prior case reports have discussed that in Type B insulin resistance presentation with DKA is rare, and that it is often associated with elevated adiponectin levels, female sex, black ethnicity, presence of acanthosis nigricans, and concomitant rheumatologic diagnoses [1–3] (Table 1). Our patient presented with DKA, lacked acanthosis nigricans and had normal adiponectin levels, therefore not the typical presentation of Type B insulin resistance. However, there is no definite set of clinical characteristics that assist in differentiating between the two subtypes [4].

From a serologic perspective, our patient had elevated insulin antibody levels but we were unable to evaluate for the presence of an insulin receptor antibody. To our knowledge, at this time a commercial assay for insulin receptor antibody remains unavailable in the United States of America. Presence of both insulin receptor antibody and a borderline elevated insulin antibody titer has been reported in one case (Table 1). Elevated plasma adiponectin levels may indicate insulin receptor dysfunction, therefore supporting a diagnosis of Type B insulin resistance; however, lack of an accepted reference standard limits the comparison of adiponectin levels attained by different laboratory assays [13].

Differentiating between Type B insulin resistance and insulin antibody-mediated insulin resistance should not deter the initiation of therapeutic planning. Kim et al. [4] were the first to report the successful use of the NIH immunomodulatory protocol developed for Type B insulin resistance in a patient with insulin antibody-mediated insulin resistance, suggesting efficacy in both autoimmune pathologies. Similarly, the excellent clinical outcome in our case supports that distinguishing the precise subtype of antibody-mediated extreme insulin resistance should not delay immunomodulatory therapy implementation. Our patient responded favorably to two cycles of the NIH immunomodulatory protocol and now has good glycemic control on metformin and pioglitazone alone.

**Figure 2:**
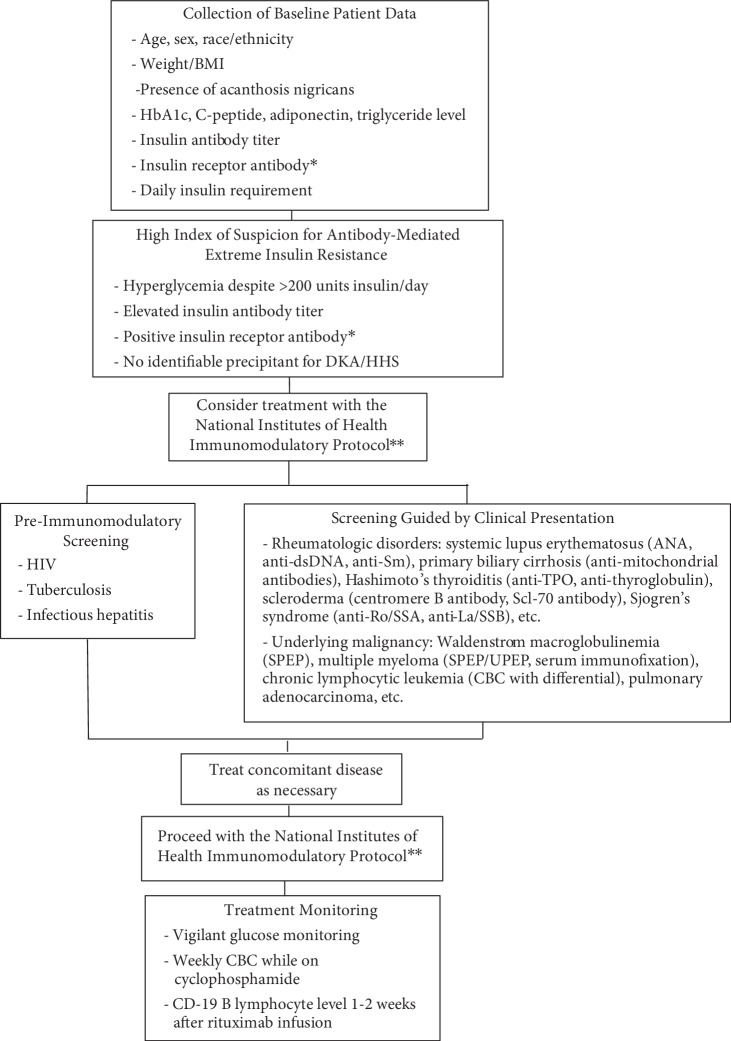
Pre-Immunomodulatory Therapy Screening Protocol. BMI = body mass index; HbA1c = glycated hemoglobin; DKA = diabetic ketoacidosis; HHS = hyperosmolar hyperglycemic state; HIV = Human Immunodeficiency Virus; ANA = antinuclear antibodies; anti-dsDNA = anti-double stranded DNA; anti-Sm = anti Smith antibody; anti-TPO = anti-thyroid peroxidase antibody; Scl-70 antibody = anti-topoisomerase 1 antibody; SPEP = serum protein electrophoresis; UPEP = urine protein electrophoresis; CBC = complete blood count. ^∗^There is no commercially available insulin receptor antibody assay in the United States of America at this time. ^∗∗^National Institutes of Health Immunomodulatory Protocol originally detailed by Malek et al. [2]. Immunotherapy cycles consist of rituximab infusion, 2 mg/mL in 0.9% sodium chloride, 750 mg/m^2^ body surface area (Day 1); dexamethasone 40 mg once daily by mouth (Day 1–4); and cyclophosphamide 100 mg once daily by mouth (Day 1 through remission).

This case adds to the literature by emphasizing the importance of pre-immunomodulatory treatment screening. Our investigation resulted in a diagnosis of latent tuberculosis, prompting initiation of isoniazid and pyridoxine in conjunction with immunomodulatory therapy. In an effort to encourage standardized data collection and enhance safe immunomodulatory administration, we drew from our own treatment experience as well as prior case reports (Table 1) to create a pre-immunomodulatory therapy screening protocol (Figure 2). Considering that cases of antibody-mediated insulin resistance have shown both associations and causal relationships with rheumatologic disorders and malignancies (Table 1), investigation into such pathologies should be guided by clinical suspicion based on a patient's presenting history, physical exam, and basic laboratory findings. We also suggest that all patients should be assessed for HIV, tuberculosis, and infectious hepatitis considering the detrimental implications of a missed diagnosis in the setting of iatrogenic immunosuppression.

In conclusion, our case reiterates the challenge of distinguishing between Type B insulin resistance and insulin antibody-mediated insulin resistance while supporting the use of the NIH immunomodulatory protocol in cases of suspected antibody-mediated insulin resistance [2–4]. Although striving to distinguish the precise subtype of antibody-mediated extreme insulin resistance should not delay therapy, pretreatment screening is indicated to diagnose possible underlying rheumatologic disorders and malignancy as well as to detect occult infectious processes that could otherwise result in deleterious health consequences. Our proposed pre-immunomodulatory therapy screening protocol has not been validated, yet we hope it will serve as a guide for future clinical encounters.

## Figures and Tables

**Table 1:** Case reports of antibody-mediated insulin resistance

	Age, sex (years, M/F)	Race/ethnicity	Acanthosis nigricans	Triglyceride (mg/dL)	HbA1c (%) [mmol/mol]	C-peptide (ng/mL)	Adiponectin (mcg/mL)	Insulin antibody titer	Insulin receptor antibody	Maximum documented insulin requirement	Associated rheumatologic/ malignancy diagnosis
Our case	56, M	Hispanic	N	85	12.4 [112]	3	18	Elevated		2526 U/day	
Malek et al, Case B-30 [2]^∗^	20, F	Black	Y	42	11.9 [107]		54.4		Y	18000 U/day	Mixed connective tissue disorder
Malek et al, Case B-33 [2]	50, F	Black		68	9 [75]		21.3		Y	1300 U/day	Systemic lupus erythematosus
Malek et al, Case B-34 [2]	62, M	Black		36	9.2 [77]		12.5		Y	1250 U/day	Systemic lupus erythematosus
Malek et al, Case B-35 [2]^∗∗^	17, F	Black	Y	41	6.8 [51]		8.3		Y	0 U/day	
Malek et al, Case B-36 [2]	64, M	Canadian		102	11.7 [104]		15.2		Y	1800 U/day	Systemic lupus erythematosus
Malek et al, Case B-37 [2]	58, F	Black		92	10.2 [88]		24.8		Y	7000 U/day	
Malek et al, Case B-38 [2]	21, F	Black		41	13.5 [124]		43.4		Y	750 U/day	
Kim et al, Case 1 [4]^∗∗∗^	63, M	Black	N	52	9.6 [81]	4.2	23	Borderline elevated	Y	21000 U/day	
Kim et al, Case 2 [4]	61, F	Jamaican	Y	39	15.5 [146]	3.03	29		Y	11650 U/day	
Kim et al, Case 3 [4]	60, M	Black	N	103	10.8 [95]	1.75	16	Elevated	N	1500 U/day	
Liminet et al. [5]	83, M		N		11–12 [97–108]	3.0		Elevated	N	100 U/hr	Lymphocytic lymphoma
Mizuhashi et al. [6]	62, M				10.4 [90]			Elevated			Pulmonary adenocarcinoma
Takaya et al. [7]	66, M				11.9 [107]	5.24		Elevated	N	>150 U/day	
Greenfield et al. [8]	68, F	White	N	442	12.4 [112]	0.98	1.7	Elevated	N	>300 U/day	
Lahtela et al. [9]	27, M				12.6 [114]	0		Elevated		360 U/day	
Rhie et al, Case 1 [10]	71, M	White						Elevated	N	440 U/day	Waldenstrom macroglobulinemia
Rhie et al, Case 2 [10]	69, M							Elevated	N	2050 U/day	Multiple myeloma
Segal et al. [11]	12, M	White			12.5 [113]	0		Elevated		6000 U/day	
Willard et al. [12]	39, F	Nigerian	Y	72	12 [108]	8.56	9		Y	>1000 U/day	Systemic lupus erythematosus

Y = yes; N = no; ^∗^Malek et al. [2] mentions 7 additional cases that are not included in this table given paucity of reported data. ^∗∗^Case B-35 uniquely with normoglycemia, but nevertheless with acanthosis nigricans, hyperinsulinemia, and positive insulin receptor antibody. ^∗∗∗^Insulin antibody titer listed as “borderline elevated” given reported titer of 0.4 U/ml and listed normal reference range 0.4 U/ml.
